# Supplementary material for: Strategic Fluorination to Achieve a Potent, Selective, Metabolically Stable, and Orally Bioavailable Inhibitor of CSNK2
Source: Molecules. 2024 Sep 2;29(17):4158. doi: 10.3390/molecules29174158 (PMC11397024; doi:10.3390/molecules29174158)
Supplement: Supplementary file 1 [file molecules-29-04158-s001.zip › molecules-3140548-supplementary.pdf]

Strategic Fluorination to Achieve a Potent, Selective, Metabolically-Stable and Orally-Bioavailable Inhibitor of CSNK2.

Han Wee Ong[1,2#*], Xuan Yang[1,2#], Jeffery L. Smith[2], Sharon Taft-Benz[1,3], Stefanie Howell[2], Rebekah J. Dickmander[1,4,5,6], Tammy M. Havener[2], Marcia K. Sanders[1,3], Jason W. Brown[7], Rafael M. Couñago[2,8], Edcon Chang[7], Andreas Krämer[9], Nathaniel J. Moorman[1,4,5], Mark Heise[1,3], Alison D. Axtman[1,2], David H. Drewry[1,2,5], Timothy M. Willson[1,2]

[1]Rapidly Emerging Antiviral Drug Development Initiative (READDI), Chapel Hill, North Carolina 27599, USA;
[2]Structural Genomics Consortium (SGC) and Division of Chemical Biology and Medicinal Chemistry, Eshelman School of Pharmacy, University of North Carolina at Chapel Hill, Chapel Hill, NC 27599, USA;
[3]Department of Genetics, University of North Carolina at Chapel Hill, Chapel Hill, North Carolina 27599, USA;
[4]Department of Microbiology & Immunology, University of North Carolina at Chapel Hill, Chapel Hill, North Carolina 27599, USA;
[5]Lineberger Comprehensive Cancer Center, University of North Carolina at Chapel Hill, Chapel Hill, North Carolina 27599, USA;
[6]Department of Chemistry, University of North Carolina at Chapel Hill, Chapel Hill, North Carolina 27599, USA;
[7]Takeda Development Center Americas, Inc., San Diego, California 92121, USA;
[8]Centro de Química Medicinal (CQMED), Centro de Biologia Molecular e Engenharia Genética (CBMEG), University of Campinas, Campinas, São Paulo, 13083-886, Brazil;
[9]SGC, Institute of Pharmaceutical Chemistry, Goethe University Frankfurt am Main, Max-von-Laue-Str. 9, 60438, Frankfurt am Main, Germany.
[#]co-first authors
*Correspondence: onghw@live.unc.edu

SUPPORTING INFORMATION

Figure S1.	S2
Figure S2.	S3
Table S1.	S4
Table S2.	S9
Table S3.	S9
NMR spectra for compound **2**.	S11
HPLC trace for compound **2**.	S13

`

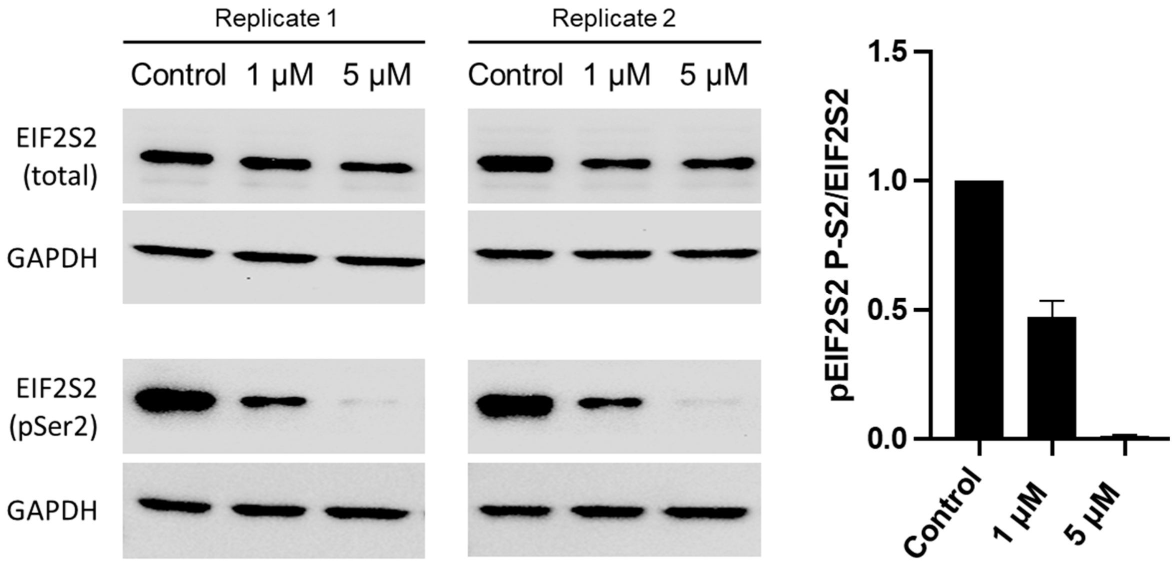

Figure S1. Phosphorylation of CSNK2 substrate EIF2S2 in A549-ACE2 cells was inhibited by 24 hour treatment of compound **2** (1 μM or 5 μM).

Acquisition Time (sec)	(0.1946, 0.0068)	***Comment***	EB7420-387-P1C DMSO 400MHZ HOESY
Date	25 Apr 2023 01:22:50		
ExpNo	3		
File Name	\\WGQDFS01\Personal\ma_xiumei\data\EB7420-387-P1C\3\PDATA\1\2rr		
Frequency (MHz)	(400.2100, 376.5269)	***Mixing Time***	0.8
Nucleus	(1H, 19F)	***Number of Transients***	8
Origin	Avance NEO Nanobay		
Original Points Count	(1024, 128)	***Owner***	nmrsu
Points Count	(1024, 1024)	***Pulse Sequence***	hoesygpph
Solvent	DMSO-d6	***Spectrum Type***	NOESY
Sweep Width (Hz)	(5258.02, 18809.57)	***Temperature (degree C)***	24.213
Title	EB7420-387-P1C DMSO 400MHZ HOESY		

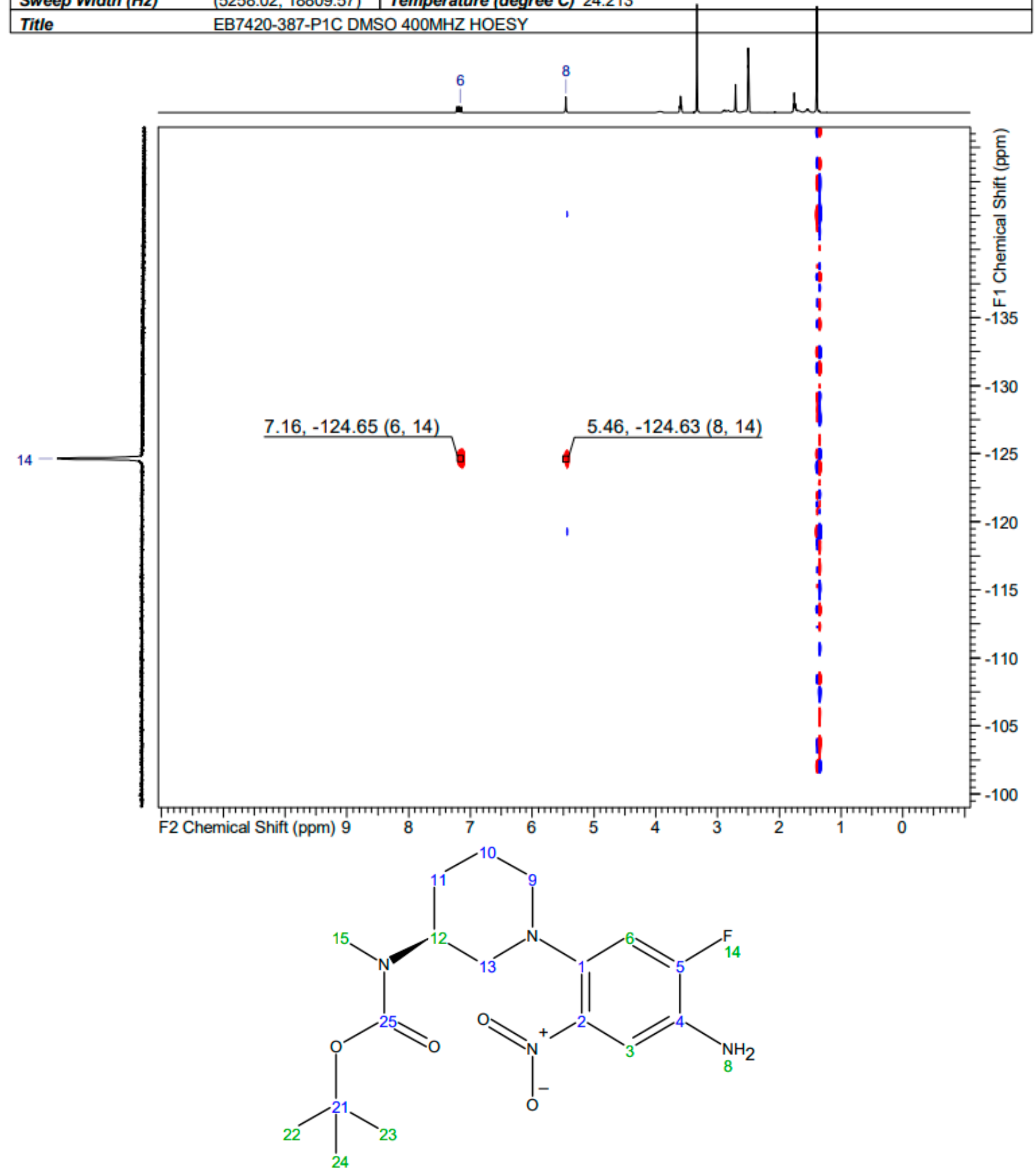

Figure S2. ^{1}H-^{19}F HOESY spectrum for compound **7**. Cross-peaks were observed between the fluorine atom (numbered 14) with the protons on the amine (numbered 8) and on the carbon numbered 6.

Table S1. Selectivity of **2** in the NanoBRET K192 Selectivity Panel.

Kinase	% occupancy at 10 μM
CSNK2A1	97.3
CSNK2A2	96.9
CLK2	84.8
CLK1	76.2
CLK4	73.8
DAPK2	72.1
DYRK1A	71.2
PHKG1	55.3
STK17B	45.5
DCLK3	44.7
CDK7 + pMB	43.9
HIPK4	39.9
MYLK4	38.5
DYRK1B	38.3
MAP4K2	31.4
PHKG2	29.7
PLK2	29.6
CDK14 + Y	29.4
CDK15 + Y	26.5
CAMK1	26.2
STK33	26.0
CDK16 + Y	25.8
HIPK3	25.5
ULK1	25.2
NEK5	24.8
MELK	24.5
MKNK2	23.6
CDK20 + H	23.4
IKBKE	23.4
CSNK1G2	21.8
HIPK2	21.5
TIE1	20.6
CDK17 + Y	20.2
CDK9 + K	19.8
MAP3K11	19.5
CDKL3	19.4
NEK11	19.1
TBK1	18.5
CAMK2D	18.4
FYN	18.1
AAK1	17.8
WEE1	17.8

BRSK1	17.7
CDK18 + Y	17.5
CDKL5	17.2
STK16	16.4
CDK2 + E1	15.9
SBK3	15.9
PTK2	15.7
PLK3	15.5
ULK2	15.5
PAK6	15.3
MAPK6	14.6
STK10	14.4
MARK2	14.2
MAP3K2	13.8
CDKL2	13.6
CDK10 + L2	13.6
BRSK2	13.6
JAK2 (V617F)	13.1
MYLK2	13.1
TNK2	12.5
CAMK2A	12.4
MET	12.3
CDK3 + E1	12.1
NLK	11.9
ITK	11.9
CDK6 + D1	11.9
FES	11.7
PRKACA	11.7
MAST4	11.4
CDK5 + CDK5R1	10.8
RPS6KA6	10.6
PRKX	10.6
FGFR1	10.4
MAPK14	10.4
MAP3K3	10.1
MAPK11	9.4
ABL2	9.4
LATS2	9.4
CSNK1D	9.4
RIPK2	9.3
IRAK4	9.2
PRKACB	9.2
ERN2	9.2
TEC	9.1

RIPK1	9.1
MAP4K5	8.5
CDK4 + D3	8.5
FGFR4	8.2
TNNI3K	8.0
NEK4	7.8
EPHA4	7.8
EPHA1	7.7
BMP2K	7.6
RON	7.6
MARK4	7.4
SRMS	7.4
EPHA6	7.3
RPS6KA1	7.1
STK4	6.9
TNK1	6.8
SLK	6.6
LIMK1	6.6
STK35	6.5
RPS6KA4	6.4
SIK1	6.4
PRKCE	6.2
CDK1 + B1	6.2
STK32B	5.9
PKMYT1	5.5
AKT2	5.3
BRAF(V600E)	5.3
STK3	5.2
TEK	5.2
MAP4K1	5.1
MAP3K4	4.9
LCK	4.9
MAP3K21	4.8
EPHB1	4.7
NTRK1v1	4.6
JAK3	4.6
TLK2	4.6
AURKC	4.5
PAK4	4.5
WEE2	4.5
PTK6	4.4
EPHB4	4.4
MAP3K9	4.3
LTK	4.2
NEK2	4.2

MERTK	4.2
STK38L	4.0
TYRO3	3.9
PLK4	3.8
PRKAA1	3.8
GAK	3.3
FER	3.1
ERN1	3.0
BTK	3.0
MAP4K3	2.9
SIK3	2.7
IRAK3	2.4
MAP3K19	2.4
RPS6KA2	2.4
MAPK9	2.4
RET	1.8
RPS6KA3	1.8
CDKL1	1.5
SGK1	1.5
CSNK1A1L	1.5
AURKB	1.4
INSR	1.2
MLTK	1.1
LIMK2	1.1
NEK9	1.0
JNK3	1.0
MAPK4	0.9
MAPK8	0.6
BMX	0.6
PRKAA2	0.5
MYLK3	0.4
STK11	0.1
NTRK2	-0.1
MAP3K12	-0.1
LATS1	-0.4
MUSK	-0.5
FGFR3	-0.7
STK38	-0.8
CHEK2	-0.8
MAST3	-0.9
LRRK2	-1.5
PTK2B	-1.6
NUAK1	-1.7
RIOK2	-1.8
NEK1	-1.9

SNRK	-2.0
MAP3K10	-2.4
EPHA7	-2.5
IGF1R	-2.6
FGFR2	-2.9
TXK	-3.2
SNF1LK2	-4.8
ICK	-6.1
ULK3v1	-7.4
STK36	-7.6
AXL	-9.2
AURKA	-9.3
NEK3	-11.1
FLT3	-13.5
TLK1	-23.1
NIM1K	-23.7

Table S2. Crystallographic refinement statistics.

**Data collection**	CSNK2A1-**2**
Beamline	I03/DLS
Space group	$P4_32_12$
Cell dimensions	
a, b, c (Å)	127.18, 127.18, 124.03
α, β, γ (°)	90, 90, 90
Resolution (Å)*	29.67-2.70 (2.83-2.70)
unique observations*	28599 (3722)
R_{pim}*	0.052 (0.536)
Completeness (%)*	100.0 (100.0)
Multiplicity*	27.6 (28.9)
mean I/σI*	13.1 (1.9)
$CC_{1/2}$*	0.998 (0.804)
**Refinement**	
R_{work} / R_{free}	0.2020 / 0.2496
No. of atoms	5673
overall B-factors (Å^2)	57.62
Rms deviations	
Bond lengths (Å)	0.005
Bond angles (°)	1.230
Ramachandran outlier (%)	0.2
**Protein Data Bank entry**	9FYF

*Values for the highest resolution shell are shown in parentheses.

Table S3. Comparison of *in vitro* metabolic clearance of compounds **1** and **2**.

**Compound**	**R**	**MLM CL_{int} (mL/min/kg)[a]**	**Mouse Hepatocytes CL_{int} (mL/min/kg)[a]**	**HLM CL_{int} (mL/min/kg)[b]**	**Human Hepatocytes CL_{int} (mL/min/kg)[b]**
**1**	H	<24[c]	143[c]	12.5	4.1
**2**	F	48.5	180	12.9	6.9

[a]Metabolism in MLM and HLM quantified by LC-MS over five time points over 1 h, scaled by scaling factors of mass of liver per body weight and microsomal concentration in liver. [b]Metabolism in hepatocytes quantified by LC-MS over 2 h, scaled by scaling factors of mass of liver per body weight and hepatocyte concentration in liver. [c]Data from ref. [1].

References

(1) Yang, X.; Ong, H. W.; Dickmander, R. J.; Smith, J. L.; Brown, J. W.; Tao, W.; Chang, E.; Moorman, N. J.; Axtman, A. D.; Willson, T. M. Optimization of 3-Cyano-7-Cyclopropylamino-Pyrazolo[1,5-a]Pyrimidines toward the Development of an In Vivo Chemical Probe for CSNK2A. *ACS Omega* **2023**, *8* (42), 39546–39561. https://doi.org/10.1021/acsomega.3c05377.

NMR spectra for compound 2.

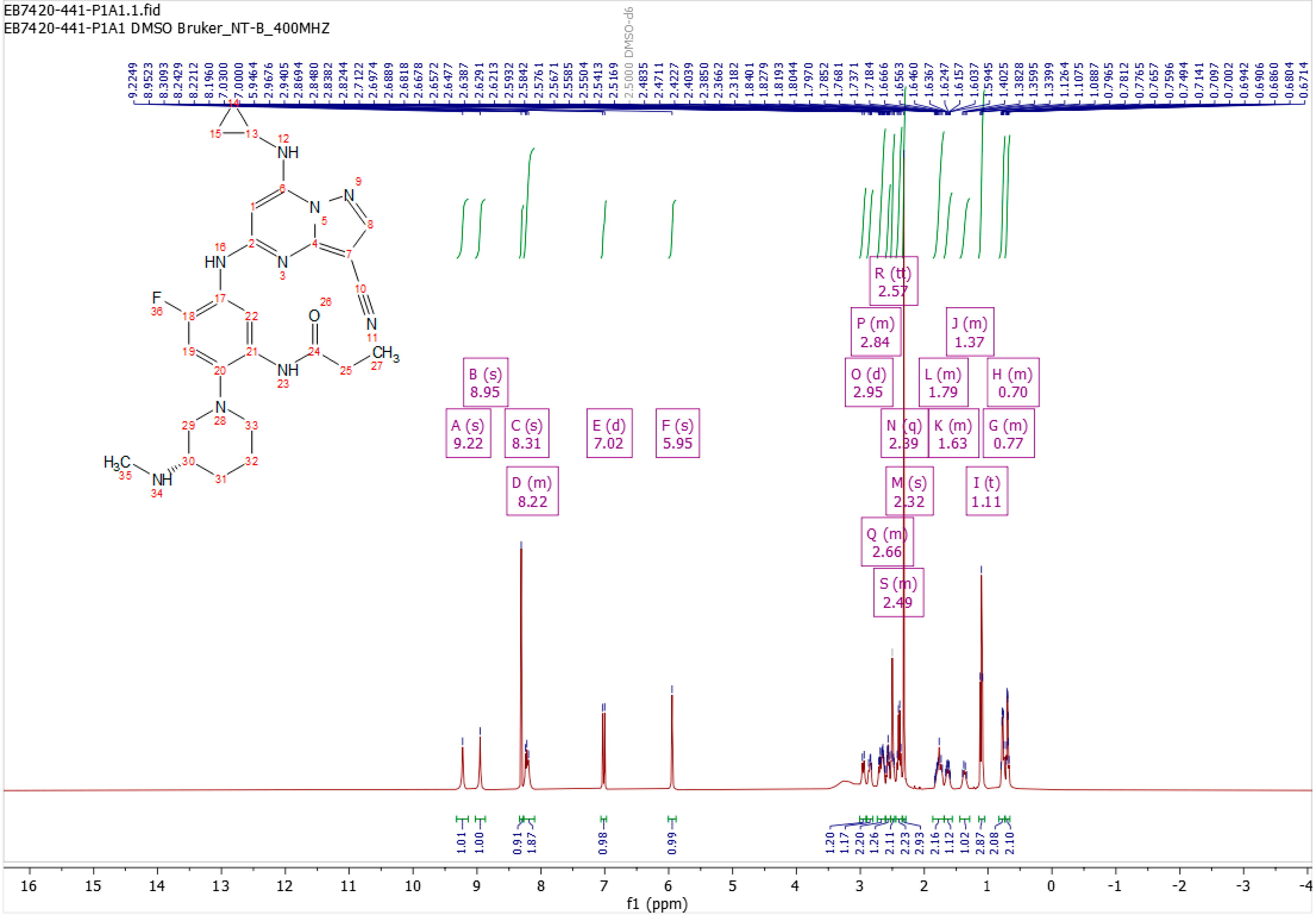

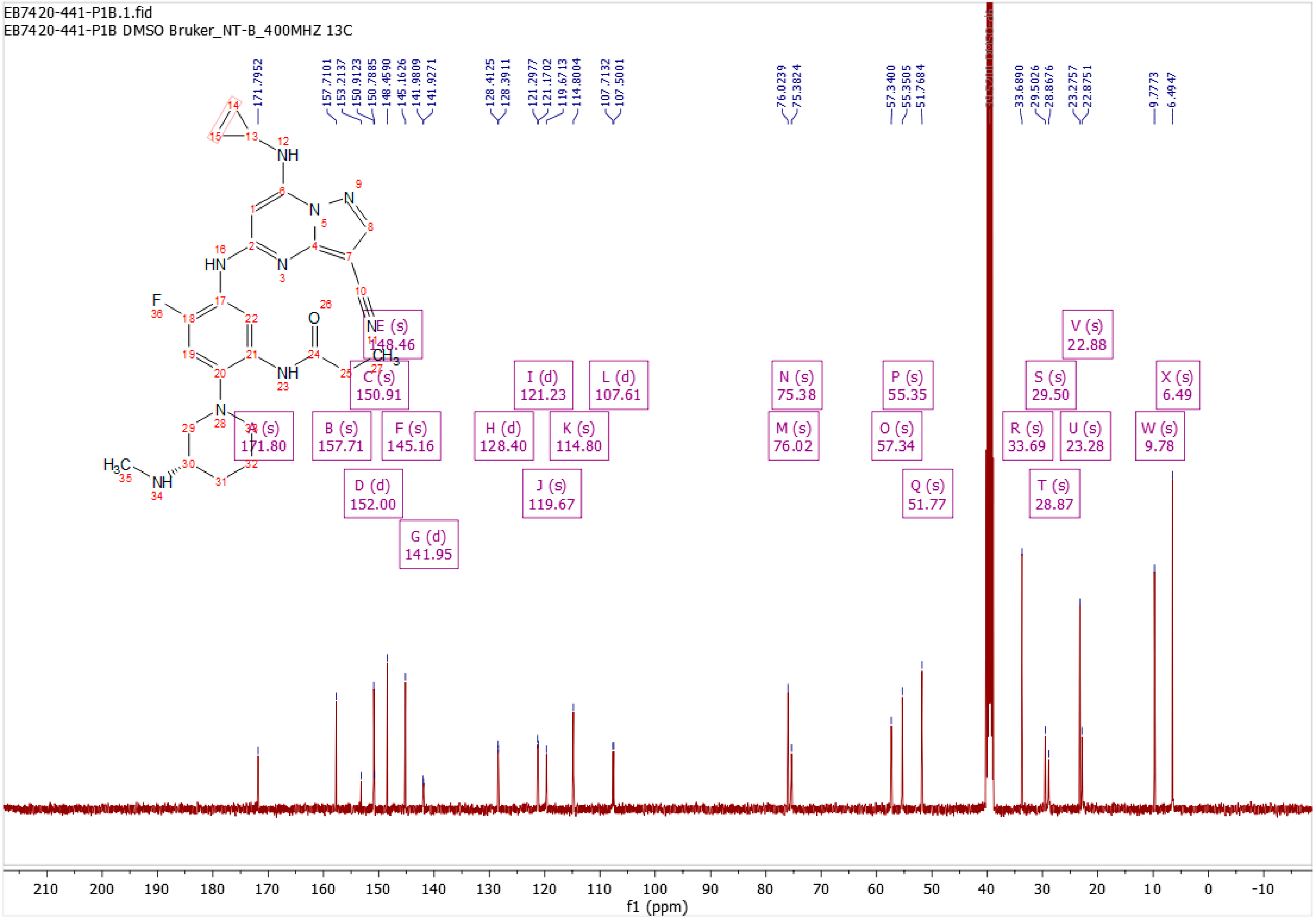
EB7420-441-P1B.1.fid
EB7420-441-P1B DMSO Bruker_NT-B_400MHZ 13C
171.7952
157.7101
153.2137
150.9123
150.7885
148.4590
145.1626
141.9809
141.9271
128.4125
128.3911
121.2977
121.1702
119.6713
114.8004
107.7132
107.5001
76.0239
75.3824
57.3400
55.3505
51.7684
33.6890
29.5026
28.8676
23.2757
22.8751
9.7773
6.4947
A (s) 171.80
B (s) 157.71
C (s) 150.91
D (d) 152.00
E (s) 148.46
F (s) 145.16
G (d) 141.95
H (d) 128.40
I (d) 121.23
J (s) 119.67
K (s) 114.80
L (d) 107.61
M (s) 76.02
N (s) 75.38
O (s) 57.34
P (s) 55.35
Q (s) 51.77
R (s) 33.69
S (s) 29.50
T (s) 28.87
U (s) 23.28
V (s) 22.88
W (s) 9.78
X (s) 6.49
F
HN
NH
N
O
CH3
H3C
f1 (ppm)

HPLC trace for compound 2.

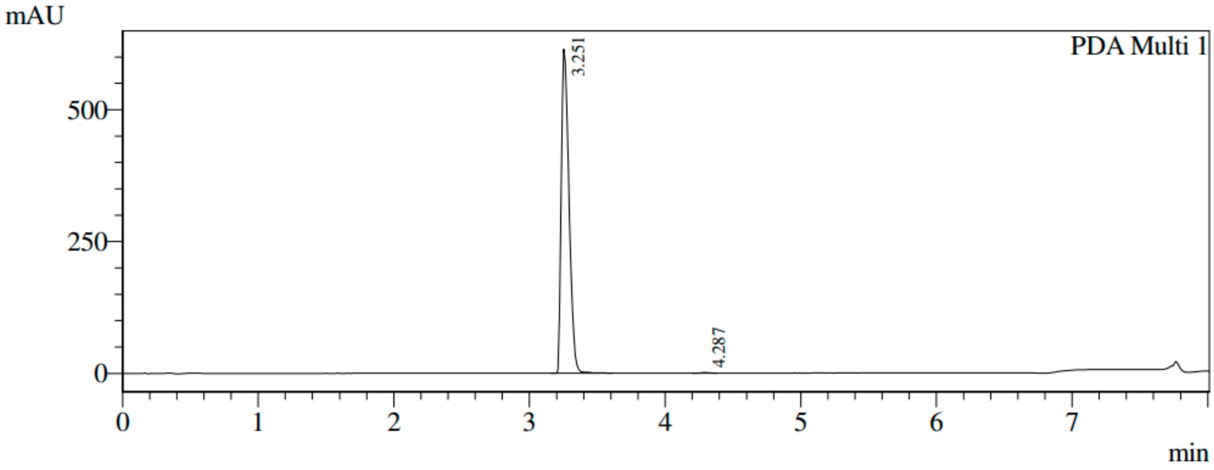

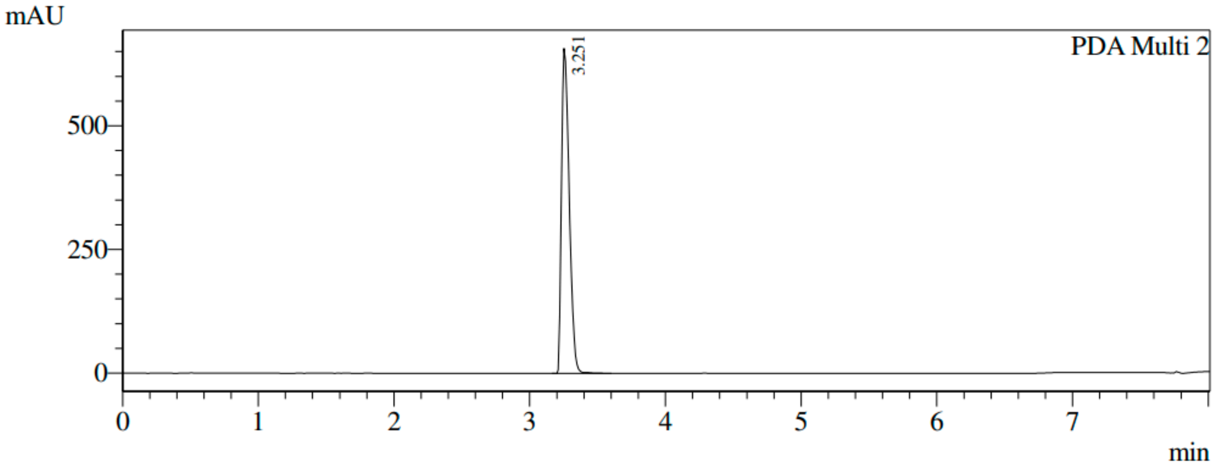

1 PDA Multi 1 / 220nm 4nm

Peak#	Ret. Time	Height	Height %	Area	Area %
1	3.251	614289	99.801	2439152	99.828
2	4.287	1225	0.199	4214	0.172
Total		615514	100.000	2443367	100.000

2 PDA Multi 2 / 254nm 4nm

Peak#	Ret. Time	Height	Height %	Area	Area %
1	3.251	656998	100.000	2611799	100.000
Total		656998	100.000	4481641	100.000
